# Abnormal Expressions of DNA Glycosylase Genes NEIL1, NEIL2, and NEIL3 Are Associated with Somatic Mutation Loads in Human Cancer

**DOI:** 10.1155/2016/1546392

**Published:** 2016-03-03

**Authors:** Kazuya Shinmura, Hisami Kato, Yuichi Kawanishi, Hisaki Igarashi, Masanori Goto, Hong Tao, Yusuke Inoue, Satoki Nakamura, Kiyoshi Misawa, Hiroyuki Mineta, Haruhiko Sugimura

**Affiliations:** ^1^Department of Tumor Pathology, Hamamatsu University School of Medicine, Hamamatsu 431-3192, Japan; ^2^Research Equipment Center, Hamamatsu University School of Medicine, Hamamatsu 431-3192, Japan; ^3^Division of Carcinogenesis and Prevention, National Cancer Center Research Institute, Tokyo 104-0045, Japan; ^4^Department of Otolaryngology/Head and Neck Surgery, Hamamatsu University School of Medicine, Hamamatsu 431-3192, Japan

## Abstract

The effects of abnormalities in the DNA glycosylases NEIL1, NEIL2, and NEIL3 on human cancer have not been fully elucidated. In this paper, we found that the median somatic total mutation loads and the median somatic single nucleotide mutation loads exhibited significant inverse correlations with the median NEIL1 and NEIL2 expression levels and a significant positive correlation with the median NEIL3 expression level using data for 13 cancer types from the Cancer Genome Atlas (TCGA) database. A subset of the cancer types exhibited reduced NEIL1 and NEIL2 expressions and elevated NEIL3 expression, and such abnormal expressions of NEIL1, NEIL2, and NEIL3 were also significantly associated with the mutation loads in cancer. As a mechanism underlying the reduced expression of NEIL1 in cancer, the epigenetic silencing of* NEIL1* through promoter hypermethylation was found. Finally, we investigated the reason why an elevated NEIL3 expression level was associated with an increased number of somatic mutations in cancer and found that NEIL3 expression was positively correlated with the expression of APOBEC3B, a potent inducer of mutations, in diverse cancers. These results suggested that the abnormal expressions of NEIL1, NEIL2, and NEIL3 are involved in cancer through their association with the somatic mutation load.

## 1. Introduction

*NEIL1* (ENSG00000140398, OMIM #608844),* NEIL2* (ENSG00000154328, OMIM #608933), and* NEIL3* (ENSG00000109674, OMIM #608934) are structural human homologues of* Escherichia coli* (*E. coli*)* Nei* and* Fpg*, the genes encoding a DNA glycosylase that initiates the base excision repair (BER) process. These three homologues also have actual functional activities as DNA glycosylases [1–5], although the modes of strand incision differ; NEIL1 and NEIL2 have strong *β*, *δ* elimination activities, but NEIL3 has only a weak *β* elimination activity [6, 7]. Regarding substrate specificity, the three DNA glycosylases have broad and overlapping specificities for modified bases. The preferred substrates for all of them are spiroiminodihydantoin and guanidinohydantoin, which are highly mutagenic DNA lesions, but various other DNA lesions are also recognized by some of them. For example, 8-hydroxyguanine, which is also a mutagenic base lesion, is a substrate for NEIL1 and NEIL2, but not for NEIL3 [7, 8]. Because of these DNA glycosylase activities, NEIL1, NEIL2, and NEIL3 also have the ability to regulate the mutation frequency in cells. Deficiencies of NEIL1 or NEIL2 in mammalian cells reportedly lead to an elevated mutation frequency [9–11], and the overproduction of mouse NEIL3 in an* E. coli fpg nei mutY* strain reduced the spontaneous mutation frequency [12]. These findings indicate that NEIL1, NEIL2, and NEIL3 have the ability to suppress mutations in cells. Therefore, NEIL1, NEIL2, and NEIL3 are important enzymes to maintain the stability of genomic DNA by preventing mutations.

Recent advances in high-throughput sequencing technology have enabled associations between specific gene abnormalities and the somatic mutation load to be investigated in human cancer. Such investigations have revealed that the inactivation of mismatch repair genes, inactivating mutations of* BRCA1* (ENSG00000012048),* BRCA2* (ENSG00000139618),* POLE* (ENSG00000177084), and* POLK* (ENSG00000122008), or the overexpression of APOBEC3B (ENSG00000179750) causes mutagenesis in cancer [13–17]. At present, however, a definitive relationship between the status of DNA glycosylases, including NEIL1, NEIL2, and NEIL3, and the extent of somatic mutations in genomic DNA has not been demonstrated. Since NEIL1, NEIL2, and NEIL3 are involved in the repair of mutagenic bases and are capable of suppressing mutations, we investigated the relationship between the expression levels of NEIL1, NEIL2, and NEIL3 and the somatic mutation load using whole-exome sequencing data derived from the Cancer Genome Atlas (TCGA) database. We found, for the first time, that the abnormal expressions of NEIL1, NEIL2, and NEIL3 are associated with somatic mutation loads in diverse cancers.

## 2. Materials and Methods

### 2.1. Collection of Publicly Available Data on Somatic Mutations, mRNA Expression, and DNA Methylation

mRNA expression, somatic mutation, and the DNA methylation data of 13 cancer types [bladder urothelial carcinoma (TCGA ID: BLCA), breast invasive carcinoma (BRCA), colon adenocarcinoma (COAD), head and neck squamous cell carcinoma (HNSC), kidney chromophobe renal cell carcinoma (KICH), kidney renal clear cell carcinoma (KIRC), kidney renal papillary cell carcinoma (KIRP), lung adenocarcinoma (LUAD), lung squamous cell carcinoma (LUSC), prostate adenocarcinoma (PRAD), rectum adenocarcinoma (READ), stomach adenocarcinoma (STAD), and thyroid carcinoma (THCA)] were collected from the TCGA data portal (https://tcga-data.nci.nih.gov/tcga/) in April 2014. The number of cases used in this study is summarized in Supplementary Table S1 in Supplementary Material available online at http://dx.doi.org/10.1155/2016/1546392. The expression data were obtained as processed RNA-sequence (RNA-seq) data in the form of RNA-seq by Expectation Maximization (RSEM) [18], excluding the RNA-seq data of STAD, which was obtained in the form of Reads Per Kilobase of Exon Model (RPKM) per million mapped reads [19]. The somatic mutation data were obtained using whole-exome sequencing and are shown in the form of a mutation annotation format (MAF) file. The DNA methylation data obtained using the HumanMethylation450 platform (Illumina Inc., CA, USA) were shown as the *β* value (ratio of the methylated probe intensity and the overall intensity). Whether the expressions of NEIL1, NEIL2, and NEIL3 are epigenetically silenced by promoter hypermethylation was determined based on the following 4 criteria, according to a previous report [13] with some modifications: (1) a mean DNA methylation *β* value at the CpG site near the transcription start site in normal tissue < 0.4; (2) a difference in the *β* value between the 90th percentile of *β* value in tumor tissue and the mean in normal tissue > 0.1; (3) a fold expression change between the mean in normal tissue and the mean of the 10% of tumor tissue with the highest *β* value > 1.5; (4) a Spearman rank correlation value between DNA methylation and gene expression < −0.25.

### 2.2. 5-Aza-Deoxycytidine (5-aza-dC) Treatment

The gastric cancer cell lines MKN45 and MKN74, which were obtained from the Human Science Research Resource Bank (Osaka, Japan), were treated with 2 *μ*M of 5-aza-dC (Sigma-Aldrich, St. Louis, MO, USA) for 48 h, as described previously [20].

### 2.3. Quantitative Reverse-Transcription- (QRT-) Polymerase Chain Reaction (PCR)

Total RNA was extracted using an RNeasy Plus Mini Kit (Qiagen, Valencia, CA, USA) and was converted to cDNA using a SuperScript First-Strand Synthesis System for RT-PCR (Invitrogen, Carlsbad, CA, USA). Real-time QRT-PCR was performed using cDNA, a set of primers, a QuantiTect SYBR Green PCR kit (Qiagen), and a LightCycler instrument (Roche, Palo Alto, CA, USA). The PCR primers were as follows: 5′-AAG TCA GGT TCT TCC GCC AC-3′ and 5′-CGG TAG GCA CTG CTC TCA AAG-3′ for the NEIL1 transcript (transcript variant 2: NM_024608), 5′-GCA GAA TAA CTG TGT GCC GCT-3′ and 5′-ACC CTG CTA GAT GTC CAA CTG ATT-3′ for the NEIL3 transcript, and 5′-GCT CAG ACA CCA TGG GGA AG-3′ and 5′-TGT AGT TGA GGT CAA TGA AGG GG-3′ for the GAPDH (ENSG00000111640) transcript. The relative amounts of NEIL1 or NEIL3 transcript were normalized to the amount of the GAPDH transcript.

### 2.4. Immunohistochemical Analysis

Paraffin embedded blocks of head and neck squamous cell carcinoma (HNSCC) cancer tissue and corresponding normal tissue from a total of 77 sporadic cases of primary HNSCC were obtained from Hamamatsu University Hospital (Japan). The mean age of the patients was 67.2 years (standard deviation: 9.4 years), and the sample included 69 men and 8 women. The sections were boiled at 96°C for 40 min in TE solution (pH 9.0) for antigen retrieval and incubated for 5 min in a 3% hydrogen peroxide solution to block endogenous peroxidase activity. Then, the sections were incubated with an anti-NEIL1 polyclonal antibody (Sigma-Aldrich) followed by an amino acid polymer conjugated with goat anti-rabbit IgG and HRP (Histofine Simple Stain MAX PO, Nichirei, Tokyo, Japan). The antigen-antibody complex was visualized with 3,3′-diaminobenzidine tetrahydrochloride and was counterstained with hematoxylin. The intensity values of the tumor cells were determined using a 3-point scale according to the color of the cells after NEIL1 immunostaining: 0: blue; 1: light brown; 2: brown. The percentage of cells with each intensity value was then multiplied by the intensity value, to obtain an immunohistochemical score of 0–200. The use of HNSCC tissues was approved by the Institutional Review Board of Hamamatsu University School of Medicine.

### 2.5. Statistical Analysis

The statistical analysis was performed using a Mann-Whitney *U* test, Spearman rank correlation test, or Wilcoxon matched pairs test. Overall survival curves were constructed using the Kaplan-Meier method, and the differences in the curves were evaluated using the log-rank test. The hazard ratio (HR) and its 95% confidence interval (CI) were calculated using the Cox proportional hazard model in both univariate and multivariate analyses. JMP version 9.0 software (SAS Institute, Cary, NC, USA) was used for all the statistical analyses. *P* values less than 0.05 were considered statistically significant.

## 3. Results

### 3.1. Correlations between the Expression Levels of NEIL1, NEIL2, and NEIL3 and the Extent of Somatic Mutation in Human Cancer

**Figure 1:**
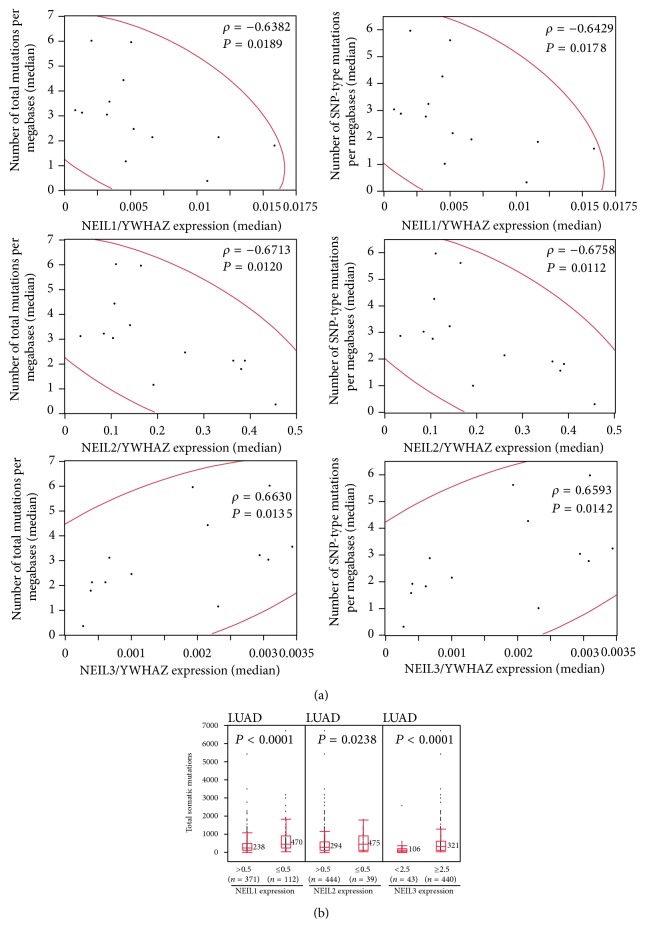
Associations between the expression levels of NEIL1, NEIL2, and NEIL3 and the somatic mutation load in human cancer. (a) Scatter plots of the median NEIL1, NEIL2, and NEIL3 expression levels and the median mutation loads in 13 cancer types, based on data from the TCGA database. The expression data for each gene was divided by that for the YWHAZ housekeeping gene. The median number of total mutations per Mb (left panels) or the median number of SNP-type mutations per Mb (right panels) was analyzed, and the Spearman rank correlation coefficient (*ρ*) and *P* values were provided. In the analysis, the prevalence of somatic mutations in exomes was calculated based on the identified mutations in the captured region. A bivariate normal ellipse (*P* = 0.95) was observed. (b) Comparison of the total somatic mutation loads between the group showing abnormal NEIL1, NEIL2, and NEIL3 expressions and the other group in lung adenocarcinoma (*n* = 483), as performed using a box-plot analysis of the data from the TCGA database. Values that were 0.5-fold the median NEIL1 expression value, 0.5-fold the median NEIL2 expression value, and 2.5-fold the median NEIL3 expression value in noncancerous lung tissue were used as the cut-off values to dichotomize the cancer cases. The *P* values (Mann-Whitney *U* test) and median mutation values are shown.

To determine whether the cancer mutation load is correlated with the expression levels of NEIL1, NEIL2, and NEIL3 in human cancer, mRNA expression data and somatic mutation data for 13 cancer types were obtained from the TCGA database. Regarding the somatic mutation data, along with the total mutation loads, SNP-type mutations, corresponding to single nucleotide exchange including synonymous and nonsynonymous mutations and not including insertion-type and deletion-type mutations, were also calculated to investigate the effects of NEIL1, NEIL2, and NEIL3 on such mutation types. Then, the median mutation loads for each cancer type and the median NEIL1, NEIL2, and NEIL3 expression values normalized to the expression value of the constitutive housekeeping gene YWHAZ (ENSG00000164924) [21] were analyzed to identify correlations. As expected, the median total mutation load and the median SNP-type mutation load showed a strong inverse correlation with the median NEIL1 expression level (*ρ* = −0.6382, *P* = 0.0189 and *ρ* = −0.6429, *P* = 0.0178, resp.) (Figure 1(a)). In addition, the median total mutation load and the median SNP-type mutation load showed a strong inverse correlation with the median NEIL2 expression level (*ρ* = −0.6713, *P* = 0.0120 and *ρ* = −0.6758, *P* = 0.0112, resp.). On the other hand, the median total mutation load and the median SNP-type mutation load showed a strong positive correlation with the median NEIL3 expression level (*ρ* = 0.6630, *P* = 0.0135 and *ρ* = 0.6593, *P* = 0.0142, resp.). Similar to above, a significant correlation was also observed when another housekeeping gene, PSMB2 (ENSG00000126067) [22, 23], was used (Supplementary Figure S1); YWHAZ and PSMB2 were used because their expression levels were usually correlated with the expressions of several other housekeeping genes in various organ tissues (Supplementary Figure S2). These results suggest that the expression levels of NEIL1, NEIL2, and NEIL3 are differentially correlated with the extent of somatic mutation in human cancer.

### 3.2. Expression Statuses of NEIL1, NEIL2, and NEIL3 and Their Associations with the Extent of Somatic Mutation in Each Cancer Type

Next, we attempted to investigate the expression statuses of NEIL1, NEIL2, and NEIL3 in each cancer type and to determine whether their abnormal expressions were associated with the mutation load of each cancer. The levels of NEIL1 and NEIL2 mRNA expression in tumor tissue, compared with normal tissue, were significantly reduced in 6 of the 13 (46.2%) cancer types and 4 of the 13 (30.8%) cancer types, respectively (Supplementary Table S2, Supplementary Figure S3). On the other hand, the level of NEIL3 mRNA expression was significantly increased in tumor tissue, compared with normal tissue, in all 13 cancer types (100%). When the 0.5-fold, 0.5-fold, and 2.5-fold values of the median expression value in noncancerous tissue samples of each organ were used as cut-off values to dichotomize the NEIL1, NEIL2, and NEIL3 expression values in the cancer cases, respectively, cancers with reduced NEIL1 expression, reduced NEIL2 expression, and elevated NEIL3 expression were detected in 31.4%, 9.0%, and 79.4% of all cancers, respectively (Supplementary Table S3). These results suggested that a subset of human cancers exhibited reduced NEIL1 and NEIL2 expressions and an elevated NEIL3 expression.

We next investigated whether the abnormal expressions of NEIL1, NEIL2, and NEIL3 were associated with the mutation load in each cancer type. The total mutation loads were significantly higher in the group of cancers with the lower NEIL1 and NEIL2 expression levels in 4 of the 13 (30.8%) cancer types and 2 of the 13 (15.4%) cancer types, respectively (Figure 1(b), Supplementary Figure S4, and Supplementary Tables S4 and S5). In addition, the total mutation loads were significantly higher in the group of cancers with the higher NEIL3 expression levels in 7 of the 13 (53.8%) cancer types (Table 1, Figure 1(b), and Supplementary Figure S4). These results suggested that the abnormal expressions of NEIL1, NEIL2, and NEIL3 are associated with the mutation load in cancer.

### 3.3. Epigenetic Silencing of NEIL1 Expression in Human Cancer

**Figure 2:**
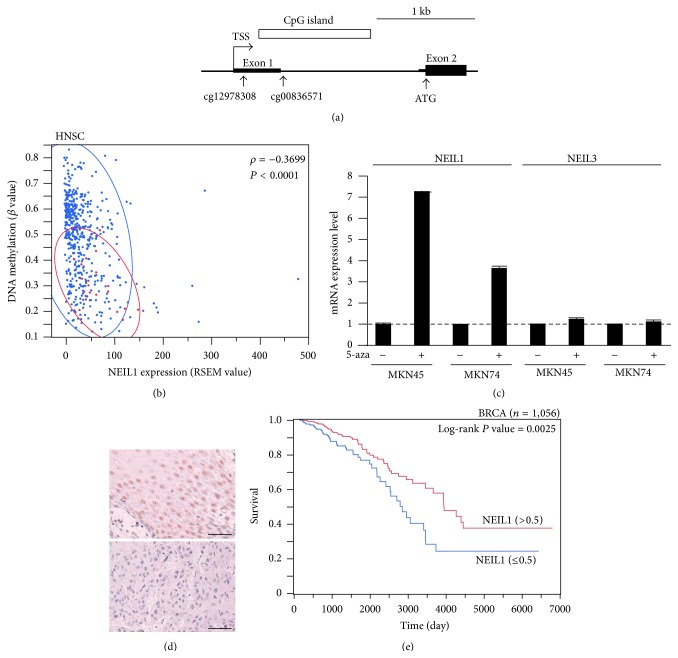
Epigenetic silencing of NEIL1 expression in human cancer. (a) Map of the DNA methylation probes near the transcription start sites (TSSs) of the* NEIL1* gene. The vertical arrows mark the position of the DNA methylation probes (CpG sites) or the translation initiation site (ATG). The thicker section in the exon region indicates the coding sequence. (b) Representative result showing the inverse correlation between DNA methylation at the* NEIL1* CpG site and NEIL1 expression in cancer. A scatter plot analysis was performed for DNA methylation at the cg12978308 probe site and the NEIL1 mRNA expression level in HNSCC using data from the TCGA database. The Spearman rank correlation coefficient (*ρ*) and *P* values were provided. A bivariate normal ellipse (*P* = 0.95) was observed for normal tissue samples (red) and cancerous tissue samples (blue). (c) Effects of 5-aza-dC on the NEIL1 and NEIL3 expression levels in gastric cancer cell lines. The cell lines were treated with 5-aza-dC, and the NEIL1 and NEIL3 expression levels were measured using a real-time QRT-PCR analysis. The amounts of NEIL1 or NEIL3 transcripts normalized to the amount of GAPDH transcript are shown in the graph. The average expression levels in untreated cells were set at 1.0. Values are the mean ± standard error of three independent experiments. (d) Downregulation of NEIL1 protein expression in primary HNSCC. Representative results for NEIL1 expression in noncancerous head and neck epithelium (upper panel) and HNSCC (lower panel) are shown. Scale bar = 50 *μ*m. (e) Impact of reduced NEIL1 expression on overall survival in primary breast cancer patients. The survival curves for breast cancer patients (*n* = 1,056) were based on data from the TCGA database and were generated using the Kaplan-Meier method. The patients were divided into two groups using a cut-off value of 0.5-fold the median NEIL1 expression value in noncancerous breast tissue. Log-rank: *P* = 0.0025.

To identify the mechanism underlying the reduction in NEIL1 and NEIL2 expression in cancer, we investigated whether these genes were epigenetically silenced in cancer using DNA methylation data from the TCGA database. Nine [breast invasive carcinoma, colon adenocarcinoma, HNSCC, clear cell renal cell carcinoma (RCC), papillary RCC, lung adenocarcinoma, lung squamous cell carcinoma, rectal adenocarcinoma, and stomach adenocarcinoma] of the 13 (69.2%) cancer types satisfied the 4 criteria for epigenetic silencing described in Section 2 for the* NEIL1* gene, whereas none of the cancer types satisfied the criteria for the* NEIL2* or* NEIL3* gene (Table 2, Figures 2(a) and 2(b), Supplementary Figure S5, and Supplementary Table S6). Together with a previous finding that the region around the transcription start site of the* NEIL1* gene exhibits promoter activity [24], these results suggest that these cancer types exhibit epigenetic silencing of the* NEIL1* via promoter hypermethylation. To confirm the possibility of* NEIL1* epigenetic silencing, we treated two gastric cancer cell lines (MKN45 and MKN74) with the cytosine methylation inhibitor 5-aza-dC and measured the levels of NEIL1 expression using QRT-PCR. The expression level of NEIL1, but not of NEIL3, was increased in both cell lines by the 5-aza-dC treatment, strengthening the notion of the epigenetic silencing of NEIL1 expression (Figure 2(c)). We next compared the level of NEIL1 protein expression between cancerous tissues and corresponding noncancerous epithelial tissues using an immunohistochemical analysis of 77 primary HNSCCs. The NEIL1 protein expression level was significantly lower in the cancerous tissues than in the noncancerous tissues (*P* < 0.0001 by Wilcoxon matched pairs test) (Figure 2(d), Supplementary Table S7). This result suggests that the level of NEIL1 protein expression is reduced in a subset of primary HNSCCs, possibly supporting the idea that the NEIL1 expression level was reduced in cancer because of promoter hypermethylation. Finally, we investigated the impact of the reduction in NEIL1 expression in cancer on the overall survival of the patients. A Kaplan-Meier analysis showed that a reduction in NEIL1 expression was associated with a poorer outcome in patients with breast invasive carcinoma (*P* = 0.0025, log-rank test) (Figure 2(e)) but not in patients with the other 12 cancer types. Moreover, a multivariate analysis using the Cox proportional hazard model showed that a reduction in NEIL1 expression was associated with a significantly elevated risk of a poor survival outcome among patients with breast invasive carcinoma (HR: 2.194; 95% CI: 1.417–3.394; *P* = 0.0005) (Supplementary Table S8). These results suggest that a reduction in NEIL1 expression is an independent predictor of a poor survival outcome among patients with breast invasive carcinoma.

### 3.4. Cooccurrence of Elevated NEIL3 and APOBEC3B Expressions in Human Cancer

Since NEIL1, NEIL2, and NEIL3 have been experimentally shown to have the ability to suppress mutations in human cells and/or in bacterial cells [9–12], the finding that the reductions in NEIL1 and NEIL2 expression were associated with the increase in the number of somatic mutations in cancer seems reasonable. However, the association between the elevation in NEIL3 expression and the increased number of somatic mutations in cancer is surprising. To clarify the reason for this association, we investigated the relationship between the expressions of NEIL3 and APOBEC3B, a known inducer of mutations [15, 16]. The APOBEC3B expression level was significantly higher in the group of cancers with a high NEIL3 expression level than in the group of cancers with a low NEIL3 expression level in 10 of the 13 (76.9%) cancer types (Table 3, Supplementary Figure S6). Moreover, a significant positive correlation was found between the NEIL3 and APOBEC3B expression levels in 10 (76.9%) cancer types (Table 3, Supplementary Figure S6). These results suggested that the expressions of NEIL3 and APOBEC3B were positively correlated in human cancer. We suspect that this correlation may explain why the elevation in NEIL3 expression was associated with an increased number of somatic mutations in cancer.

## 4. Discussion

Using data for 13 cancer types from the TCGA database, we revealed that the median somatic total and SNP-type mutation loads exhibited significant inverse correlations with the median NEIL1 and NEIL2 expression levels and a significant positive correlation with the median NEIL3 expression level. We also showed that a subset of human cancers exhibited reduced NEIL1 and NEIL2 expression levels and an elevated NEIL3 expression level, and these abnormal expressions of NEIL1, NEIL2, and NEIL3 were associated with the mutation load in cancer. We then showed that the reduced NEIL1 expression level observed in various cancers was due to epigenetic silencing by promoter hypermethylation and that such reduction was an independent predictor of a poor outcome among patients with breast invasive carcinoma. Finally, NEIL3 expression was shown to be correlated with the expression of APOBEC3B, a potent inducer of mutations, possibly explaining why an increased NEIL3 expression level was associated with the somatic mutation load in cancer. Thus, our results suggest that the abnormal regulation of NEIL1, NEIL2, and NEIL3 expression is involved in the development of cancer via an increase in the prevalence of somatic mutations, providing a new and important link between abnormalities in the DNA glycosylases NEIL1, NEIL2, and NEIL3 and human cancer.

Using a TCGA-based analysis, associations between abnormal NEIL1, NEIL2, or NEIL3 expressions and the somatic mutation load were apparently demonstrated in various cancer types for the first time. The association between reductions in NEIL1 and NEIL2 expressions and the increased number of somatic mutations in cancer is understandable, but the association between an elevation in NEIL3 expression and an increased number of somatic mutations in cancer seems surprising at first glance, since NEIL1, NEIL2, and NEIL3 all have the ability to suppress mutations [9–12]. The upregulation of NEIL3 expression in diverse cancer types is consistent with the results of a previous report by Hildrestrand et al. [25], but the effect of such upregulation on cancer has not yet been determined. Our demonstration of a correlation between NEIL3 expression and APOBEC3B expression may explain why an increase in NEIL3 expression is associated with the somatic mutation load, since APOBEC3B is involved in mutagenesis in multiple distinct human cancers [15, 16]. Although the precise mechanism was not investigated in the present study, we speculated that since the effect of APOBEC3B on the increase in mutations may be greater than the effect of NEIL3 on a decrease in mutations through its DNA glycosylase activity, the coelevated expressions of NEIL3 and APOBEC3B may lead to the observed increase in the number of somatic mutations in cancer. Alternatively, NEIL3 might be involved in APOBEC3B-induced mutagenesis. Further investigation of such issues is needed.

In this study, we found 9 cancer types that showed epigenetic silencing of the* NEIL1* gene via promoter hypermethylation using data from the TCGA database. Among them, the epigenetic silencing of NEIL1 expression in HNSCC, lung adenocarcinoma, lung squamous cell carcinoma, colon adenocarcinoma, and rectal adenocarcinoma was consistent with the findings of previous reports [24, 26, 27], whereas the findings in the remaining 4 cancer types, that is, breast invasive carcinoma, clear cell RCC, papillary RCC, and stomach adenocarcinoma, were novel findings. Although further experiments, such as 5-aza-dC treatment and a NEIL1 protein expression analysis for each of the latter 4 cancer types, are needed to determine the epigenetic silencing of the* NEIL1* gene via promoter hypermethylation in these cancer types, we suspect that the epigenetic silencing of the* NEIL1* gene via promoter hypermethylation might be the chief mechanism underlying the downregulation of NEIL1 expression in diverse human cancers. Interestingly, in breast invasive carcinoma, which is one of the cancers that shows the epigenetic silencing of* NEIL1*, a reduction in NEIL1 expression was shown to be an independent predictor of a poor survival outcome. This novel finding may be useful for the management of breast cancer patients, and if this marker is used in conjunction with other prognosis markers, such as the hormone receptor status [28], the management of breast cancer patients could be further improved. Regarding this point, in our preliminary analysis using data from the TCGA database, combinations of the NEIL1 mRNA expression level and either the hormone receptor status or the HER2 status were shown to be excellent prognostic markers (Supplementary Figure S7). Since a reduction in NEIL1 expression was associated with an increased somatic mutation level and mutations in cancer-associated genes can lead to the exaggeration of the malignant potential, such as an increase in the proliferation rate, this kind of phenotypic change might explain the difference in survival outcomes between patients with and those without a reduction in NEIL1 expression.

So far, several forms of germline nonsynonymous* NEIL1* or* NEIL2* mutations have been experimentally demonstrated to actually have reduced or absent repair activity [10, 11, 29, 30]. Human cells containing such* NEIL1* or* NEIL2* mutations are considered to have a reduced capacity to repair mutagenic bases; thus, similar to cancers with a reduced NEIL1 or NEIL2 expression levels, a higher incidence of mutation is likely to occur in the cells, leading to cancer susceptibility. This scenario is compatible with a previous paper reporting a germline* NEIL2* variant that is a marker for risk and the progression of squamous cell carcinomas of the oral cavity and oropharynx [31] and that is selectively found in familial colorectal cancer patients, but not in healthy controls [32]. Future genome-wide analyses of cancers derived from individuals with germline* NEIL1* or* NEIL2* mutations should clarify the role of NEIL1 and NEIL2 in the prevention of mutations.

In this study, the 0.5-fold, 0.5-fold, and 2.5-fold values of the median expression value in noncancerous tissue samples of each organ were used as cut-off values to dichotomize the NEIL1, NEIL2, and NEIL3 expression values in the cancer cases, respectively. If an expression level is downregulated or upregulated in a disease, a fold-change value of 0.5 and 2.5, respectively, has been used to dichotomize disease cases in previous reports [33, 34]; therefore, these values were used in our analysis.

In conclusion, our study indicates that the abnormal expressions of NEIL1, NEIL2, and NEIL3 are likely to be involved in mutagenesis in human cancer. Since little is known about gene abnormalities identified by whole-exome sequencing data that induce mutations in cancer, our findings regarding these novel mutagenic factors should contribute to our general understanding of human cancer.

## Supplementary Material

Supplementary Table S1: Sample size of TCGA dataset used in this study.
Supplementary Table S2: Abnormal NEIL1, NEIL2, and NEIL3 expressions in human cancer.
Supplementary Table S3: Incidence of the cancer cases showing abnormal NEIL1, NEIL2, or NEIL3 expression.
Supplementary Table S4: Associations between reduced NEIL1 expression levels and increased numbers of somatic mutations in human cancers.
Supplementary Table S5: Associations between reduced NEIL2 expression levels and increased numbers of somatic mutations in human cancers.
Supplementary Table S6: List of DNA methylation sites used for the analysis of epigenetic silencing of the NEIL1, NEIL2, and NEIL3 genes.
Supplementary Table S7: Immunohistochemical score of NEIL1 protein in head and neck squamous cell carcinoma.
Supplementary Table S8: Cox proportional hazard analysis of potential predictors of a poor prognosis in breast invasive carcinoma patients (n = 1,056) using data from the TCGA database.
Supplementary Figure S1: Scatter plots of the median NEIL1, NEIL2, and NEIL3 expression levels and the median mutation loads in 13 cancer types.
Supplementary Figure S2: Correlations of PSMB2 and YWHAZ expressions with the expressions of other housekeeping genes in various organs.
Supplementary Figure S3: Representative results of abnormal NEIL1, NEIL2, and NEIL3 expressions in human cancer.
Supplementary Figure S4: Comparison of the total somatic mutation loads between the group showing abnormal NEIL1, NEIL2, and NEIL3 expressions and the other group in various carcinomas, as performed using a box-plot analysis.
Supplementary Figure S5: Inverse correlation between DNA methylation at the NEIL1 CpG site and NEIL1 expression in various human cancers.
Supplementary Figure S6: Strong positive relationship between NEIL3 expression and APOBEC3B expression in human cancer.
Supplementary Figure S7: Impact of reduced NEIL1 expression in conjunction with hormone receptor status or HER2 status on overall survival in primary breast cancer patients.

## Figures and Tables

**Table 1:** Associations between elevated NEIL3 expression levels and increased numbers of somatic mutations in human cancers.

Organ	TCGA ID	Number of cases	Mann-Whitney *U* test			Spearman rank correlation	
			Grouped by NEIL3 expression level (<2.5/≥2.5 or <10/≥10^a^)		*P* ^c^ (“increase” or “decrease” in mutation number)	Rho	*P* ^d^
			Median mutation number per sample^b^	Number of cases			
Urinary bladder	BLCA	129	122/**236**	8/121	0.0341 (**increase**)	0.1593	0.0714
Breast	BRCA	977	30/**45**	62/915	<0.0001 (**increase**)	0.3006	**<0.0001**
Colon	COAD	209	127/**146.5**	83/126	0.0942	0.1121	0.1061
Head and neck	HNSC	489	151/**167.5**	203/286	0.0477 (**increase**)	0.0019	0.9673
Kidney	KICH	66	81.5/**92**	28/38	0.3705	0.2637	**0.0324**
Kidney	KIRC	212	**387**/86.5	12/200	0.0770	0.1179	0.0867
Kidney	KIRP	168	**92**/87.5	20/148	0.7409	0.0117	0.8800
Lung	LUAD	483	106/**321**	43/440	<0.0001 (**increase**)	0.3287	**<0.0001**
Lung	LUSC	179	196.5/**316**	8/171	0.0133 (**increase**)	0.2112	**0.0045**
Prostate	PRAD	258	50/**60.5**	76/182	0.0012 (**increase**)	0.3030	**<0.0001**
Rectum	READ	81	113.5/**123**	32/49	0.2707	0.2900	**0.0086**
Stomach	STAD	224	92/**187**	76/148	<0.0001 (**increase**)	0.5105	**<0.0001**
Thyroid gland	THCA	404	9/**10**	220/184	0.1612	0.0690	0.1664

^a^A value 2.5-fold the median NEIL3 expression value in noncancerous tissue samples of each organ was used as the cut-off value to dichotomize the cancer cases. In the LUSC cases, a value 10-fold the median NEIL3 expression value in noncancerous lung tissue samples was used.

^b^Higher numbers of median somatic mutation per sample are shown in bold face.

^c^A Mann-Whitney *U* test was used to perform the statistical analysis. If the *P* value was less than 0.05, indicating a significant change, a significant “increase” or “decrease” in the number of somatic mutations per sample was shown.

^d^If significant (less than 0.05), the *P* value was shown in bold face.

**Table 2:** Epigenetic silencing of NEIL1 expression in human cancer.

Organ (TCGA ID)	cg number (CpG site ID)	DNA methylation level			Gene expression level		DNA methylation level and gene expression level		
		Number of cases (N/T)	Difference in the *β* value between the 90th percentile of *β* value in tumor tissue and the mean in normal tissue	Percentage of the hypermethylated tumors^a^	Number of cases (N/T )	Fold expression change between the mean in normal tissue and the mean of the 10% of tumor tissue with the highest *β* value	Number of cases (N/T)	Spearman rank correlation value between DNA methylation and gene expression	
								Rho	*P*
Breast (BRCA)	12978308	96/745	0.109	7.2%	69/723	2.039	69/723	−0.3729	<0.0001
Colon (COAD)	12978308	38/301	0.291	29.9%	19/250	2.087	19/250	−0.5241	<0.0001
Head and neck (HNSC)	12978308	50/529	0.323	57.7%	20/498	2.354	20/498	−0.3699	<0.0001
Kidney (KIRC)	00836571	160/325	0.110	7.1%	24/303	2.407	24/303	−0.4755	<0.0001
Kidney (KIRP)	12978308	45/226	0.351	21.2%	23/182	2.276	23/182	−0.6219	<0.0001
Lung (LUAD)	12978308	32/465	0.109	64.3%	21/422	2.249	21/422	−0.3695	<0.0001
Lung (LUSC)	12978308	42/359	0.254	27.0%	8/358	1.624	8/358	−0.2799	<0.0001
Rectum (READ)	12978308	7/99	0.145	11.1%	2/90	1.998	2/90	−0.3699	0.0003
Stomach (STAD)	12978308	2/325	0.290	40.3%	0/231	2.208^b^	0/231	−0.5465	<0.0001

Epigenetic silencing of the *NEIL1* gene was determined according to the following four criteria: (1) a mean *β* value in normal tissue < 0.4; (2) a difference in the *β* value between the 90th percentile of *β* value in tumor tissue and the mean in normal tissue > 0.1; (3) a fold expression change between the mean in normal tissue and the mean of the 10% of tumor tissue with the highest *β* value > 1.5; (4) a Spearman rank correlation value between DNA methylation and gene expression < −0.25. This table includes only the cancers that fulfilled these four criteria.

^a^Percentage of cancers with the following *β* value: the mean *β* value in normal tissue + more than 0.15.

^b^Since there is no normal tissue with DNA methylation data in STAD, the mean expression value of 33 gastric normal tissues used in Supplementary Table S2 was utilized for the calculation.

**Table 3:** Associations between NEIL3 and APOBEC3B expression levels in human cancer.

Organ	TCGA ID	Number of cases	Mann-Whitney *U* test			Spearman rank correlation	
			Grouped by NEIL3 expression level (<2.5/≥2.5^a^)		*P* ^c^ (“increase” or “decrease” in APOBEC3B expression)	Rho	*P* ^d^
			Median APOBEC3B expression^b^	Number of cases			
Urinary bladder	BLCA	241	146/**382**	18/223	0.0034 (**increase**)	0.0697	0.2811
Breast	BRCA	1056	45/**175**	76/980	<0.0001 (**increase**)	0.5215	**<0.0001**
Colon	COAD	260	169/**231**	108/152	0.0191 (**increase**)	0.1569	**0.0113**
Head and neck	HNSC	498	**501**/466	209/289	0.2122	0.2326	**<0.0001**
Kidney	KICH	66	132/**149**	28/38	0.5463	0.1312	0.2935
Kidney	KIRC	519	26.9/**65.1**	31/488	<0.0001 (**increase**)	0.5777	**<0.0001**
Kidney	KIRP	198	33.1/**55**	22/176	0.0017 (**increase**)	0.4466	**<0.0001**
Lung	LUAD	490	60.4/**159**	43/447	<0.0001 (**increase**)	0.2759	**<0.0001**
Lung	LUSC	490	195/**496**	5/485	0.0187 (**increase**)	0.1302	**0.0039**
Prostate	PRAD	333	19/**32.9**	97/236	<0.0001 (**increase**)	0.5876	**<0.0001**
Rectum	READ	92	172/**247**	36/56	0.0985	0.1559	0.1379
Stomach	STAD	238	2.75/**3.92**	79/159	0.0007 (**increase**)	0.1848	**0.0042**
Thyroid gland	THCA	508	37.2/**66.5**	276/232	<0.0001 (**increase**)	0.4242	**<0.0001**

^a^A value 2.5-fold the median NEIL3 expression value in noncancerous tissue samples of each organ was used as the cut-off value to dichotomize the cancer cases.

^b^Higher numbers of median APOBEC3B expression values are shown in bold face. RPKM value was used to show expression level in stomach cancer; on the other hand RSEM value was used in the other organs' cancers.

^c^A Mann-Whitney *U* test was used to perform the statistical analysis. If the *P* value was less than 0.05, indicating a significant change, a significant “increase” or “decrease” in the APOBEC3B expression was shown.

^d^If significant (less than 0.05), the *P* value was shown in bold face.
