# Supplementary material for: Abnormal Expressions of DNA Glycosylase Genes NEIL1, NEIL2, and NEIL3 Are Associated with Somatic Mutation Loads in Human Cancer
Source: Oxid Med Cell Longev. 2016 Mar 3;2016:1546392. doi: 10.1155/2016/1546392 (PMC4794593; doi:10.1155/2016/1546392)
Supplement: Supplementary file 1 — Supplementary Table S1: Sample size of TCGA dataset used in this study. Supplementary Table S2: Abnormal NEIL1, NEIL2, and NEIL3 expressions in human cancer. Supplementary Table S3: Incidence of the cancer cases showing abnormal NEIL1, NEIL2, or NEIL3 expression. Supplementary Table S4: Associations between reduced NEIL1 expression levels and increased numbers of somatic mutations in human cancers. Supplementary Table S5: Associations between reduced NEIL2 expression levels and increased numbers of somatic mutations in human cancers. Supplementary Table S6: List of DNA methylation sites used for the analysis of epigenetic silencing of the NEIL1, NEIL2, and NEIL3 genes. Supplementary Table S7: Immunohistochemical score of NEIL1 protein in head and neck squamous cell carcinoma. Supplementary Table S8: Cox proportional hazard analysis of potential predictors of a poor prognosis in breast invasive carcinoma patients (n = 1,056) using data from the TCGA database. Supplementary Figure S1: Scatter plots of the median NEIL1, NEIL2, and NEIL3 expression levels and the median mutation loads in 13 cancer types. Supplementary Figure S2: Correlations of PSMB2 and YWHAZ expressions with the expressions of other housekeeping genes in various organs. Supplementary Figure S3: Representative results of abnormal NEIL1, NEIL2, and NEIL3 expressions in human cancer. Supplementary Figure S4: Comparison of the total somatic mutation loads between the group showing abnormal NEIL1, NEIL2, and NEIL3 expressions and the other group in various carcinomas, as performed using a box-plot analysis. Supplementary Figure S5: Inverse correlation between DNA methylation at the NEIL1 CpG site and NEIL1 expression in various human cancers. Supplementary Figure S6: Strong positive relationship between NEIL3 expression and APOBEC3B expression in human cancer. Supplementary Figure S7: Impact of reduced NEIL1 expression in conjunction with hormone receptor status or HER2 status on overall survival in primar [file 1546392.f1.docx]

Supplementary Information

**Abnormal expressions of DNA glycosylase genes NEIL1, NEIL2, and NEIL3 are associated with somatic mutation loads in human cancer**

**Supplementary Tables**

1. **Supplementary Table S1:** Sample size of TCGA dataset used in this study
2. **Supplementary Table S2:** Abnormal NEIL1, NEIL2, and NEIL3 expressions in human cancer
3. **Supplementary Table S3:** Incidence of the cancer cases showing abnormal NEIL1, NEIL2, or NEIL3 expression
4. **Supplementary Table S4:** Associations between reduced NEIL1 expression levels and increased numbers of somatic mutations in human cancers
5. **Supplementary Table S5:** Associations between reduced NEIL2 expression levels and increased numbers of somatic mutations in human cancers
6. **Supplementary Table S6:** List of DNA methylation sites used for the analysis of epigenetic silencing of the *NEIL1*, *NEIL2*, and *NEIL3* genes
7. **Supplementary Table S7:** Immunohistochemical score of NEIL1 protein in head and neck squamous cell carcinoma
8. **Supplementary Table S8:** Cox proportional hazard analysis of potential predictors of a poor prognosis in breast invasive carcinoma patients (*n* = 1,056) using data from the TCGA database

**Supplementary Figures**

1. **Supplementary Figure S1:** Scatter plots of the median NEIL1, NEIL2, and NEIL3 expression levels and the median mutation loads in 13 cancer types
2. **Supplementary Figure S2:** Correlations of PSMB2 and YWHAZ expressions with the expressions of other housekeeping genes in various organs
3. **Supplementary Figure S3:** Representative results of abnormal NEIL1, NEIL2, and NEIL3 expressions in human cancer
4. **Supplementary Figure S4:** Comparison of the total somatic mutation loads between the group showing abnormal NEIL1, NEIL2, and NEIL3 expressions and the other group in various carcinomas, as performed using a box-plot analysis
5. **Supplementary Figure S5:** Inverse correlation between DNA methylation at the *NEIL1* CpG site and NEIL1 expression in various human cancers
6. **Supplementary Figure S6:** Strong positive relationship between NEIL3 expression and APOBEC3B expression in human cancer
7. **Supplementary Figure S7:** Impact of reduced NEIL1 expression in conjunction with hormone receptor status or HER2 status on overall survival in primary breast cancer patients

Supplementary Table S1. Sample size of TCGA dataset used in this study

| Organ | TCGA ID | RNA expression  (N/T) | Somatic mutation (T) | DNA methylation (N/T) | Both RNA expression and somatic mutation (T) | Both RNA expression and DNA methylation (N/T) |
| --- | --- | --- | --- | --- | --- | --- |
| Urinary bladder | BLCA | 19/241 | 130 | 21/358 | 129 | 17/240 |
| Breast | BRCA | 98/1,056 | 991 | 96/745 | 977 | 69/723 |
| Colon | COAD | 41/260 | 269 | 38/301 | 209 | 19/250 |
| Head and neck | HNSC | 43/498 | 509 | 50/529 | 489 | 20/498 |
| Kidney | KICH | 25/66 | 66 | 0/66 | 66 | 0/66 |
| Kidney | KIRC | 72/519 | 235 | 160/325 | 212 | 24/303 |
| Kidney | KIRP | 30/198 | 171 | 45/226 | 168 | 23/182 |
| Lung | LUAD | 58/490 | 561 | 32/465 | 483 | 21/422 |
| Lung | LUSC | 50/490 | 178 | 42/359 | 179 | 8/358 |
| Prostate | PRAD | 50/333 | 261 | 49/340 | 258 | 35/333 |
| Rectum | READ | 9/92 | 116 | 7/99 | 81 | 2/90 |
| Stomach | STAD | 33/238 | 289 | 2/325 | 224 | 0/231 |
| Thyroid gland | THCA | 57/508 | 406 | 56/515 | 404 | 49/507 |

Supplementary Table S2. Abnormal NEIL1, NEIL2, and NEIL3 expressions in human cancer

| Organ | TCGA ID | No. (N/T) | NEIL1 expression | |  | NEIL2 expression | |  | NEIL3 expression | |
| --- | --- | --- | --- | --- | --- | --- | --- | --- | --- | --- |
|  |  |  | Median^a^, N/T | *P* (“Up” or “Down”)^b^ |  | Median, N/T | *P* (“Up” or “Down”) |  | Median, N/T | *P* (“Up” or “Down”) |
| Urinary bladder | BLCA | 19/241 | 111/**120**^c^ | 0.2513 |  | **393**/267 | 0.0008  (**Down**) |  | 4.08/**58.6** | <0.0001 (**Up**) |
| Breast | BRCA | 98/1,056 | **143**/101 | <0.0001  (**Down**) |  | **448**/397 | 0.0004  (**Down**) |  | 3.42/**52.9** | <0.0001 (**Up**) |
| Colon | COAD | 41/260 | **99.0**/84.3 | 0.0053  (**Down**) |  | 203/**348** | <0.0001 (**Up**) |  | 31.6/**85.7** | <0.0001 (**Up**) |
| Head and neck | HNSC | 43/498 | **61.6**/25.8 | <0.0001  (**Down**) |  | **389**/295 | <0.0001  (**Down**) |  | 44.1/**104** | <0.0001 (**Up**) |
| Kidney | KICH | 25/66 | **195**/66.8 | <0.0001  (**Down**) |  | **371**/358 | 0.7931 |  | 1.36/**4.13** | <0.0001 (**Up**) |
| Kidney | KIRC | 72/519 | **78.7**/67.5 | 0.0247  (**Down**) |  | **398**/328 | <0.0001  (**Down**) |  | 1.07/**12.2** | <0.0001 (**Up**) |
| Kidney | KIRP | 30/198 | **146**/136 | 0.7949 |  | 416/**434** | 0.4497 |  | 0.61/**6.45** | <0.0001 (**Up**) |
| Lung | LUAD | 58/490 | 113/113 | 0.7051 |  | **362/**340 | 0.4190 |  | 3.27/**42.4** | <0.0001 (**Up**) |
| Lung | LUSC | 50/490 | **121**/59.9 | <0.0001  (**Down**) |  | **333**/317 | 0.1146 |  | 3.02/**92.1** | <0.0001 (**Up**) |
| Prostate | PRAD | 50/333 | 136/**195** | <0.0001 (**Up**) |  | 471/**484** | 0.9154 |  | 1.11/**4.62** | <0.0001 (**Up**) |
| Rectum | READ | 9/92 | 78.2/**84.1** | 0.8162 |  | 216/**264** | 0.0787 |  | 28.1/**84.2** | 0.0003  (**Up**) |
| Stomach | STAD | 33/238 | **2.08**/1.88 | 0.1794 |  | 2.46/**5.19** | <0.0001 (**Up**) |  | 0.31/**1.08** | <0.0001 (**Up**) |
| Thyroid gland | THCA | 57/508 | 132/**142** | 0.3637 |  | **583**/571 | 0.5702 |  | 1.51/**3.52** | 0.0006 (**Up**) |

^a^RPKM value was used to show expression level in stomach cancer, on the other hand RSEM value was used in the other organ’s cancers.

^b^If the *P* value was less than 0.05, indicating a significant change, up-regulation or down-regulation of the expression in cancer was shown as “Up” or “Down”, respectively.

^c^Higher numbers of median somatic mutation per sample are shown in bold face.

Supplementary Table S3. Incidence of the cancer cases showing abnormal NEIL1, NEIL2, or NEIL3 expression

| Organ | TCGA ID | No. of cases | NEIL1 expression | |  | NEIL2 expression | |  | NEIL3 expression | |
| --- | --- | --- | --- | --- | --- | --- | --- | --- | --- | --- |
|  |  |  | No. (>0.5/<0.5)^a^ | % of the <0.5 group |  | No. (>0.5/<0.5)^a^ | % of the <0.5 group |  | No. (<2.5/>2.5)^a^ | % of the >2.5 group |
| Urinary bladder | BLCA | 241 | 194/47 | 19.5 |  | 173/68 | 28.2 |  | 18/223 | 92.5 |
| Breast | BRCA | 1,056 | 714/342 | 32.4 |  | 952/104 | 9.8 |  | 76/980 | 92.8 |
| Colon | COAD | 260 | 213/47 | 18.1 |  | 256/4 | 1.5 |  | 108/152 | 58.5 |
| Head and neck | HNSC | 498 | 66/432 | 86.7 |  | 368/130 | 26.1 |  | 209/289 | 58.0 |
| Kidney | KICH | 66 | 17/49 | 74.2 |  | 58/8 | 12.1 |  | 28/38 | 57.6 |
| Kidney | KIRC | 519 | 387/132 | 25.4 |  | 476/43 | 8.3 |  | 31/488 | 94.0 |
| Kidney | KIRP | 198 | 156/42 | 21.2 |  | 194/4 | 2.0 |  | 22/176 | 88.9 |
| Lung | LUAD | 490 | 376/114 | 23.3 |  | 451/39 | 8.0 |  | 43/447 | 91.2 |
| Lung | LUSC | 490 | 241/249 | 50.8 |  | 464/26 | 5.3 |  | 5/485 | 99.0 |
| Prostate | PRAD | 333 | 322/11 | 3.3 |  | 319/14 | 4.2 |  | 97/236 | 70.9 |
| Rectum | READ | 92 | 80/12 | 13.0 |  | 87/5 | 5.4 |  | 36/56 | 60.9 |
| Stomach | STAD | 238 | 184/54 | 22.7 |  | 237/1 | 0.4 |  | 79/159 | 66.8 |
| Thyroid gland | THCA | 508 | 473/35 | 6.9 |  | 504/4 | 0.8 |  | 276/232 | 45.7 |
| total |  | 4989 | 3423/1566 | 31.4 |  | 4539/450 | 9.0 |  | 1028/3961 | 79.4 |

^a^The 0.5-fold, 0.5-fold, and 2.5-fold values of the median expression value in non-cancerous tissue samples of each organ were used as cut-off values to dichotomize the NEIL1, NEIL2, and NEIL3 expression values in the cancer cases, respectively.

Supplementary Table S4. Associations between reduced NEIL1 expression levels and increased numbers of somatic mutations in human cancers

| Organ | TCGA ID | No. of cases | Mann-Whitney *U* test | | |
| --- | --- | --- | --- | --- | --- |
|  |  |  | Grouped by NEIL1 expression level (>0.5/<0.5^a^) | | *P*^c^  (“Increase” or “Decrease” in mutation number) |
|  |  |  | Median mutation number per sample^b^ | No. of cases |  |
| Urinary bladder | BLCA | 129 | **243**/178 | 97/32 | 0.2356 |
| Breast | BRCA | 977 | 39/**54** | 647/330 | <0.0001  (**Increase**) |
| Colon | COAD | 209 | 132/**170.5** | 169/40 | 0.0137  (**Increase**) |
| Head and neck | HNSC | 489 | **179.5**/158 | 66/423 | 0.2091 |
| Kidney | KICH | 66 | **94**/85 | 17/49 | 0.3670 |
| Kidney | KIRC | 212 | 87/87 | 157/55 | 0.7680 |
| Kidney | KIRP | 168 | **88**/87 | 127/41 | 0.0727 |
| Lung | LUAD | 483 | 238/**470.5** | 371/112 | <0.0001  (**Increase**) |
| Lung | LUSC | 179 | 280/**319** | 53/126 | 0.7104 |
| Prostate | PRAD | 258 | 56/**64** | 247/11 | 0.2552 |
| Rectum | READ | 81 | 118.5/**133** | 72/9 | 0.5679 |
| Stomach | STAD | 224 | 131/**199.5** | 172/52 | 0.0009  (**Increase**) |
| Thyroid gland | THCA | 404 | 9/**10.5** | 370/34 | 0.7398 |

^a^A value 0.5-fold the median NEIL1 expression value in non-cancerous tissue samples of each organ was used as the cut-off value to dichotomize the cancer cases.

^b^Higher numbers of median somatic mutation per sample are shown in bold face.

^c^A Mann-Whitney *U* test was used to perform the statistical analysis. If the *P* value was less than 0.05, indicating a significant change, a significant “increase” or “decrease” in the number of somatic mutations per sample was shown.

Supplementary Table S5. Associations between reduced NEIL2 expression levels and increased numbers of somatic mutations in human cancers

| Organ | TCGA ID | No. of cases | Mann-Whitney *U* test | | | |
| --- | --- | --- | --- | --- | --- | --- |
|  |  |  | Grouped by NEIL2 expression level (>0.5/<0.5^a^) | | | *P*^c^  (“Increase” or “Decrease” in mutation number) |
|  |  |  | Median mutation number per sample^b^ | | No. of cases |  |
| Urinary bladder | BLCA | 129 | 217/**225** | 92/37 | | 0.9026 |
| Breast | BRCA | 977 | 42/**58** | 877/100 | | <0.0001  (**Increase**) |
| Colon | COAD | 209 | **141.5**/122 | 206/3 | | 0.5640 |
| Head and neck | HNSC | 489 | **161**/155 | 362/127 | | 0.3322 |
| Kidney | KICH | 66 | 82/**102** | 58/8 | | 0.4318 |
| Kidney | KIRC | 212 | 86/**107** | 197/15 | | 0.1459 |
| Kidney | KIRP | 168 | 87/**117** | 165/3 | | 0.4723 |
| Lung | LUAD | 483 | 294/**475** | 444/39 | | 0.0238  (**Increase**) |
| Lung | LUSC | 179 | 299/**437.5** | 171/8 | | 0.0531 |
| Prostate | PRAD | 258 | 56/**63** | 247/11 | | 0.1354 |
| Rectum | READ | 81 | 119.5/**139** | 78/3 | | 0.2993 |
| Stomach | STAD | 224 | **156**/79 | 223/1 | | 0.2930 |
| Thyroid gland | THCA | 404 | 9.5/**14.5** | 402/2 | | 0.2113 |

^a^A value 0.5-fold the median NEIL2 expression value in non-cancerous tissue samples of each organ was used as the cut-off value to dichotomize the cancer cases.

^b^Higher numbers of median somatic mutation per sample are shown in bold face.

^c^A Mann-Whitney *U* test was used to perform the statistical analysis. If the *P* value was less than 0.05, indicating a significant change, a significant “increase” or “decrease” in the number of somatic mutations per sample was shown.

Supplementary Table S6. List of DNA methylation sites used for the analysis of epigenetic silencing of the *NEIL1*, *NEIL2*, and *NEIL3* genes

| Gene | CpG site (ID) | Distance from the transcription start site |  | Location^a^ |
| --- | --- | --- | --- | --- |
| NEIL1 | cg05281061 | -841 |  | 5' shore |
| NEIL1 | cg25938347 | -168 |  | 5' shore |
| NEIL1 | cg19831197 | -53 |  | 5' shore |
| NEIL1 | cg12600197 | 8 |  | 5' shore |
| NEIL1 | cg12978308 | 102 |  | 5' shore |
| NEIL1 | cg00836571 | 494 |  | CpG island |
| NEIL1 | cg27209457 | 817 |  | CpG island |
| NEIL1 | cg06784436 | 1,166 |  | CpG island |
| NEIL1 | cg07928073 | 1,411 |  | CpG island |
| NEIL2 | cg11908155 | -1,408 |  | 5' shore |
| NEIL2 | cg04541228 | -1,289 |  | 5' shore |
| NEIL2 | cg20207567 | -662 |  | 5' shore |
| NEIL2 | cg00873037 | -188 |  | 5' shore |
| NEIL2 | cg05267444 | -160 |  | 5' shore |
| NEIL2 | cg08325212 | -153 |  | 5' shore |
| NEIL2 | cg14033944 | -114 |  | 5' shore |
| NEIL2 | cg14684581 | -48 |  | CpG island |
| NEIL2 | cg26752888 | 108 |  | CpG island |
| NEIL2 | cg19560979 | 301 |  | CpG island |
| NEIL2 | cg06498398 | 308 |  | CpG island |
| NEIL2 | cg00598335 | 358 |  | CpG island |
| NEIL2 | cg14329976 | 640 |  | 3' shore |
| NEIL2 | cg09768257 | 1,174 |  | 3' shore |
| NEIL2 | cg17955761 | 1,258 |  | 3' shore |
| NEIL3 | cg14022913 | -1,340 |  | 5' shore |
| NEIL3 | cg10683527 | -385 |  | 5' shore |
| NEIL3 | cg09475757 | -140 |  | CpG island |
| NEIL3 | cg22833147 | -133 |  | CpG island |
| NEIL3 | cg13996392 | -66 |  | CpG island |
| NEIL3 | cg09089644 | -60 |  | CpG island |
| NEIL3 | cg05367790 | -57 |  | CpG island |
| NEIL3 | cg03237356 | -48 |  | CpG island |
| NEIL3 | cg01203443 | -19 |  | CpG island |
| NEIL3 | cg00883150 | 6 |  | CpG island |
| NEIL3 | cg19570979 | 50 |  | CpG island |
| NEIL3 | cg13743031 | 253 |  | CpG island |
| NEIL3 | cg12624080 | 452 |  | 3' shore |

^a^5′ shore: CpG site at a CpG island “shore” at the 5′ end; 3′ shore: CpG site at a CpG island “shore” at the 3′ end.

Supplementary Table S7. Immunohistochemical score of NEIL1 protein in head and neck squamous cell carcinoma (*n* = 77)

| Case No. | Age | Sex^a^ | Immunohistochemical score | |  | Case No. | Age | Sex | Immunohistochemical score | |
| --- | --- | --- | --- | --- | --- | --- | --- | --- | --- | --- |
|  |  |  | N | T |  |  |  |  | N | T |
| 1 | 63 | M | 80 | 0 |  | 40 | 64 | M | 0 | 0 |
| 2 | 65 | F | 150 | 0 |  | 41 | 70 | F | 0 | 0 |
| 3 | 65 | M | 0 | 0 |  | 42 | 68 | M | 70 | 0 |
| 4 | 57 | M | 150 | 180 |  | 43 | 58 | M | 80 | 0 |
| 5 | 76 | M | 0 | 0 |  | 44 | 71 | M | 90 | 0 |
| 6 | 84 | M | 0 | 0 |  | 45 | 46 | M | 100 | 0 |
| 7 | 80 | M | 120 | 0 |  | 46 | 75 | M | 0 | 0 |
| 8 | 67 | M | 30 | 0 |  | 47 | 80 | M | 0 | 0 |
| 9 | 45 | M | 30 | 0 |  | 48 | 56 | M | 0 | 0 |
| 10 | 61 | M | 80 | 30 |  | 49 | 71 | M | 0 | 0 |
| 11 | 75 | M | 30 | 0 |  | 50 | 73 | M | 40 | 0 |
| 12 | 90 | M | 20 | 0 |  | 51 | 60 | M | 0 | 0 |
| 13 | 45 | M | 80 | 120 |  | 52 | 76 | M | 0 | 0 |
| 14 | 82 | M | 80 | 80 |  | 53 | 74 | M | 0 | 0 |
| 15 | 79 | M | 30 | 0 |  | 54 | 75 | M | 30 | 0 |
| 16 | 67 | M | 20 | 0 |  | 55 | 61 | M | 20 | 0 |
| 17 | 55 | M | 60 | 0 |  | 56 | 54 | M | 80 | 0 |
| 18 | 65 | M | 70 | 0 |  | 57 | 74 | F | 20 | 0 |
| 19 | 53 | M | 120 | 150 |  | 58 | 58 | M | 20 | 0 |
| 20 | 63 | M | 20 | 0 |  | 59 | 76 | M | 0 | 0 |
| 21 | 64 | M | 0 | 0 |  | 60 | 78 | M | 0 | 0 |
| 22 | 45 | M | 80 | 30 |  | 61 | 63 | M | 20 | 0 |
| 23 | 68 | M | 80 | 0 |  | 62 | 67 | M | 0 | 0 |
| 24 | 71 | M | 0 | 0 |  | 63 | 57 | M | 80 | 100 |
| 25 | 66 | M | 0 | 0 |  | 64 | 78 | M | 0 | 0 |
| 26 | 70 | M | 40 | 0 |  | 65 | 75 | M | 80 | 30 |
| 27 | 66 | M | 50 | 0 |  | 66 | 65 | M | 0 | 0 |
| 28 | 71 | F | 0 | 0 |  | 67 | 63 | M | 50 | 30 |
| 29 | 72 | M | 0 | 0 |  | 68 | 52 | M | 0 | 0 |
| 30 | 68 | M | 80 | 0 |  | 69 | 72 | M | 0 | 0 |
| 31 | 63 | M | 80 | 0 |  | 70 | 71 | M | 50 | 0 |
| 32 | 72 | M | 30 | 0 |  | 71 | 71 | M | 50 | 0 |
| 33 | 64 | F | 20 | 0 |  | 72 | 76 | F | 0 | 0 |
| 34 | 69 | M | 0 | 0 |  | 73 | 62 | M | 30 | 0 |
| 35 | 68 | M | 0 | 0 |  | 74 | 56 | M | 50 | 30 |
| 36 | 66 | F | 20 | 0 |  | 75 | 83 | M | 100 | 80 |
| 37 | 63 | M | 80 | 70 |  | 76 | 80 | M | 80 | 0 |
| 38 | 72 | F | 0 | 0 |  | 77 | 64 | M | 80 | 0 |
| 39 | 68 | M | 80 | 30 |  |  |  |  |  |  |

^a^M, male; F, female.

Supplementary Table S8. Cox proportional hazard analysis of potential predictors of a poor prognosis in breast invasive carcinoma patients (*n* = 1,056) using data from the TCGA database

| Variable | Univariate analysis | |  | Multivariate analysis | |
| --- | --- | --- | --- | --- | --- |
|  | HR [95% CI]^a^ | *P* value |  | HR [95% CI] | *P* value |
| Age |  |  |  |  |  |
| >60 | 1.563 [1.054-2.329] | 0.0262 |  | 1.853 [1.219-2.838] | 0.0039 |
| <60 | 1 |  |  |  |  |
| pT factor |  |  |  |  |  |
| pT3, pT4 | 1.224 [0.751-1.924] | 0.4060 |  |  |  |
| pT1, pT2 | 1 |  |  |  |  |
| pN factor |  |  |  |  |  |
| pN1-pN3 | 2.260 [1.456-3.604] | 0.0002 |  | 1.692 [0.990-2.896] | 0.0544 |
| pN0 | 1 |  |  | 1 |  |
| Stage |  |  |  |  |  |
| III, IV | 2.022 [1.314-3.069] | 0.0016 |  | 1.604 [0.967-2.684] | 0.0675 |
| I, II | 1 |  |  | 1 |  |
| NEIL1 expression^b^ |  |  |  |  |  |
| <0.5 | 1.826 [1.224-2.714] | 0.0034 |  | 2.194 [1.417-3.394] | 0.0005 |
| >0.5 | 1 |  |  | 1 |  |

^a^HR, hazard ratio; CI, confidence interval.

^b^A cut-off value of 0.5-fold the median NEIL1 expression value in non-cancerous breast tissue was used.

**Supplementary Figure S1:** Scatter plots of the median NEIL1, NEIL2, and NEIL3 expression levels and the median mutation loads in 13 cancer types

The expression data for each gene was divided by that for the PSMB2 housekeeping gene. The median number total mutations per Mb (left panels) or the median number SNP-type mutations per Mb (right panels) was analyzed, and the Spearman rank correlation coefficient (ρ) and *P* values were provided. In the analysis, the prevalence of somatic mutations in exomes was calculated based on the identified mutations in the captured region. A bivariate normal ellipse (*P* = 0.95) was observed.

**Supplementary Figure S2:** Correlations of PSMB2 and YWHAZ expressions with the expressions of other housekeeping genes in various organs

The Spearman rank correlation test was performed between PSMB2 or YWHAZ expression level and the expressions of other housekeeping genes in normal tissues of various organs using data from the TCGA database. If the *P* value was less than 0.05, indicating a significant correlation, the value was provided. This figure includes only the organs in which the number of non-cancerous tissue samples was more than 35. The data for the kidney and lung was obtained from the KIRC and LUAD data, respectively, because their sample numbers were the highest. Gene name (ENSEMBL ID): PSMB2 (ENSG00000126067), YWHAZ (ENSG00000164924), B2M (ENSG00000166710), HPRT1 (ENSG00000165704), PPIA (ENSG00000196262), RPLP0 (ENSG00000089157), RPLP1 (ENSG00000137818).

**Supplementary Figure S3:** Representative results of abnormal NEIL1, NEIL2, and NEIL3 expressions in human cancer

A box-plot analysis was performed for NEIL1, NEIL2, and NEIL3 mRNA expressions between the non-cancerous head and neck tissues (N) and the head and neck squamous cell carcinomas (T) using data from the TCGA database. A Mann-Whitney *U* test was used to perform the statistical analysis. The down-regulation of NEIL1 and NEIL2 mRNA expression and the up-regulation of NEIL3 mRNA expression in head and neck squamous cell carcinoma are shown.

**Supplementary Figure S4:** Comparison of the total somatic mutation loads between the group showing abnormal NEIL1, NEIL2, and NEIL3 expressions and the other group in various carcinomas, as performed using a box-plot analysis

The number of total somatic mutations identified by exome sequencing was compared between the group showing abnormal NEIL1 (A), NEIL2 (B), and NEIL3 (C) expressions and the other group using the data from the TCGA database. Values that were 0.5-fold the median NEIL1 expression value, 0.5-fold the median NEIL2 expression value, and 2.5-fold (or 10-fold) the median NEIL3 expression value in non-cancerous tissue were used as the cut-off values to dichotomize the cancer cases. The *P* values (Mann-Whitney *U* test) and median mutation values are shown. Only significant results are provided; all the analytical data are summarized in Table 1, Supplementary Table S4, and Supplementary Table S5. With regards to the results of box-plot analysis for the LUAD data, they are shown in Figure 1B.

**Supplementary Figure S5:** Inverse correlation between DNA methylation at the *NEIL1* CpG site and NEIL1 expression in various human cancers

A scatter plot analysis was performed for DNA methylation at the cg12978308 or cg00836571 probe site and the NEIL1 mRNA expression level in breast invasive carcinoma (TCGA ID: BRCA), colon adenocarcinoma (COAD), kidney renal clear cell carcinoma (KIRC), kidney renal papillary cell carcinoma (KIRP), lung adenocarcinoma (LUAD), lung squamous cell carcinoma (LUSC), rectum adenocarcinoma (READ), and stomach adenocarcinoma (STAD) using data from the TCGA database. The Spearman rank correlation coefficient (ρ) and *P* values were provided. A bivariate normal ellipse (*P* = 0.95) was observed for normal tissue samples (red) and cancerous tissue samples (blue).

**Supplementary Figure S6:** Strong positive relationship between NEIL3 expression and APOBEC3B expression in human cancer

(A) Detection of the up-regulation of APOBEC3B mRNA expression in cancers showing an elevated NEIL3 expression level using data from the TCGA database. Patients with breast invasive carcinoma (upper panel), clear cell renal cell carcinoma (middle panel), and prostatic adenocarcinoma (lower panel) were divided into an elevated NEIL3 expression group (NEIL3 expression value in cancerous tissue > 2.5-fold the median NEIL3 expression value in non-cancerous tissue samples of each organ) and another group, and a box-plot analysis for APOBEC3B mRNA expression was then performed. A Mann-Whitney *U* test was used to perform the statistical analysis. (B) Detection of a positive correlation between NEIL3 expression and APOBEC3B expression in human cancer using data from the TCGA database. A scatter plot analysis was performed for NEIL3 expression and APOBEC3B expression in breast invasive carcinoma (upper panel), clear cell renal cell carcinoma (middle panel), and prostatic adenocarcinoma (lower panel). The Spearman rank correlation coefficient (ρ) and *P* values were provided, and a bivariate normal ellipse (*P* = 0.95) was observed. (C) Detection of a positive correlation between NEIL3 expression and APOBEC3B expression in human cancers other than ones shown in (B). A scatter plot analysis was performed for NEIL3 expression and APOBEC3B expression in colon adenocarcinoma (TCGA ID: COAD), head and neck squamous cell carcinoma (HNSC), kidney renal papillary cell carcinoma (KIRP), lung adenocarcinoma (LUAD), lung squamous cell carcinoma (LUSC), stomach adenocarcinoma (STAD), and thyroid carcinoma (THCA). The Spearman rank correlation coefficient (ρ) and *P* values were provided, and a bivariate normal ellipse (*P* = 0.95) was observed.

**Supplementary Figure S7:** Impact of reduced NEIL1 expression in conjunction with hormone receptor status or HER2 status on overall survival in primary breast cancer patients

The survival curves for breast cancer patients were based on data from the TCGA database and were generated using the Kaplan–Meier method. The patients were divided into two groups using a cut-off value of 0.5-fold the median NEIL1 expression value in non-cancerous breast tissue and either of estrogen receptor (ER) status (A), progesterone receptor (PgR) status (B), or HER2 status (C). Statuses of ER, PgR, and HER2 were determined by immunohistochemical analysis.
